# Resilience of Neural Networks Underlying the Stroop Effect in the Aftermath of Severe COVID-19: fMRI Pilot Study

**DOI:** 10.3390/brainsci15060635

**Published:** 2025-06-12

**Authors:** Valérie Beaud, Nicolas Farron, Eleonora Fornari, Vincent Dunet, Sonia Crottaz-Herbette, Stephanie Clarke

**Affiliations:** 1 Service Universitaire de Neuroréhabilitation, CHUV, Centre Hospitalier Universitaire Vaudois, 1005 Lausanne, Switzerland; valerie.beaud@psychologie.ch (V.B.); sonia.crottaz-herbette@chuv.ch (S.C.-H.); 2 Service de Radiodiagnostic et Radiologie Interventionnelle, CHUV, Centre Hospitalier Universitaire Vaudois, 1005 Lausanne, Switzerland; eleonora.fornari@chuv.ch (E.F.);

**Keywords:** severe COVID-19, brain plasticity, fatigue, Stroop task, fMRI

## Abstract

Background: Alterations in resting-state functional connectivity and in activation patterns elicited during cognitive tasks were reported in acute to chronic stages of mild, moderate and critical SARS-CoV-2 infection, suggesting the dysregulation of specialised neural networks. In this pilot study, we report on activation patterns elicited by the colour–word Stroop task in patients who suffered from severe COVID-19 requiring Intensive Care Unit hospitalisation but who had no prior or COVID-19-related brain damage. Methods: Neural activity elicited during a 16 min long colour–word Stroop task was investigated with 3T fMRI 9 months after severe SARS-CoV-2 infection in six patients and in twenty-four control subjects. Results: Patients’ performance in the Stroop task was within normal limits, with the exception of one (out of six) response time in one patient and one (out of six) accuracy measure in another patient. Activation elicited by the Stroop effect, i.e., the contrasting Incongruent vs. Congruent condition, differed between the first and second parts of the task. In controls, the Stroop effect yielded an increase in activity in prefrontal, cingulate and parieto-temporal clusters as well as in the nucleus accumbens during the first part, and the activity receded during the second part in most regions. Two distinct response profiles were found among patients: (i) a Stroop effect-linked increase during the first part followed by a partial decrease during the second part, as in healthy subjects; and (ii) a weak or absent Stroop effect increase during the first part followed by a partial increase during the second part. Conclusions: The normal performance presented by patients on the Stroop task was associated with two distinct activation patterns. They may represent different resilience profiles of the corresponding neural networks and be indicative of propensity for further recovery and/or susceptibility to therapeutic interventions.

## 1. Introduction

The post-acute and early chronic stages of severe COVID-19 are often marked by fatigue, fatigability, multidomain complaints and cognitive deficits, as reported in nu1merous studies [1,2,3,4,5,6,7,8,9,10,11,12,13,14,15,16,17,18,19,20], even in patients who did not suffer COVID-19-related stroke and/or cardiac arrest and who had no prior history of neurological or psychiatric disease or cognitive dysfunction [2,21].

COVID-19 is often associated with the dysregulation of neural networks, as demonstrated in a series of reports of resting-state functional connectivity as well as in a study of task-related functional connectivity and two studies of patterns of neural activity elicited by cognitive tasks. A recent systematic review highlighted functional alterations of prefrontal and parahippocampal regions, with an enhancement of resting-state functional connectivity in the frontal, temporal and anterior piriform cortex and a reduction in the cerebellum, orbitofrontal cortex and middle temporal gyrus. Task-related functional connectivity, assessed with the Stroop colour–word task, was shown to be increased in long COVID [22]. Patterns of neural activity were investigated using working memory tasks, and individual studies documented that in comparison to control subjects, greater activation across the working memory network in post-COVID-19 condition [23] and stronger, bilateral activation of the middle frontal gyrus were found in patients suffering from post-COVID-19 fatigue [24].

Thus, current evidence documents alterations of resting-state and of task-related functional connectivity as well as of activation patterns after mild to severe COVID-19 (for references, see above). Both increases and decreases in resting-state connectivity appear to affect networks known to be involved in cognitive functions. Several studies suggest more marked alterations after moderate and severe than after mild COVID-19 or report correlations with cognitive symptoms [25,26,27] and post-COVID-19 condition. It remains, however, unclear how far the dysregulation of functional connectivity reflects the initial severity of the SARS-CoV-2 infection, the time since disease onset or interindividual differences. The latter may be due to differences in type, extent and location of nervous tissue damage and may be indicative of a propensity for further recovery or of susceptibility to specific therapeutic interventions.

In this pilot fMRI activation study, we examined a homogeneous population of patients who suffered from severe COVID-19 and required Intensive Care Unit hospitalisation. All patients were included before the introduction of COVID-19 vaccination and investigated 9 months after the onset of SARS-CoV2 infection. None of the patients had prior or COVID-19-related brain damage. We used the colour–word Stroop fMRI paradigm to explore interindividual differences in the resilience of the underlying neural networks. The Stroop task consists of indicating the colour of test items in three conditions—Incongruent (ink colour is different from word meaning), Congruent (ink colour is the same as word meaning), and Neutral (ink colour of rectangles) [28]. It has been repeatedly used for fMRI investigations in healthy subjects [29]. The ability to perform the Stroop task is known to rely on several neural networks, involving the bilaterally inferior frontal gyrus, anterior cingulate cortex, insula, intraparietal sulcus and superior and inferior parietal lobules, as well as the occipital gyri [30]. This activation pattern tends to remain stable across healthy aging with the exception of a tendency for greater frontal involvement in elderly subjects [31].

**Figure 1:**
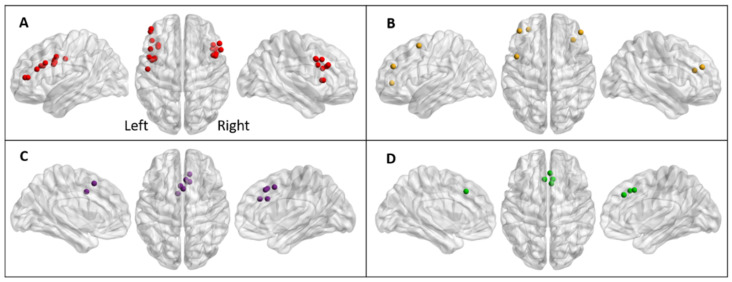
Regions implicated in the cascade-of-control model of the Stroop effect [29]. (**A**) Regions involved in establishing a bias towards task-relevant sensory or perceptual information (red dots). (**B**) Regions maintaining the relevant information in working memory (yellow dots). (**C**) Regions involved in response selection (purple dots). (**D**) Regions involved in response evaluation and feedback (green dots). Lateral and upper views of the right and left hemispheres. Coloured dots mark coordinates of significant effects as described in prior studies (Supplemental Table S1).

The interference between the task-relevant and task-irrelevant information is believed to occur at different levels, involving a cascade of control. M. T. Banich based her model on a series of neuroimaging studies, mostly in healthy subjects, contrasting the Incongruent with the Congruent or control condition of the Stroop task (Figure 1 and Supplemental Table S1). The first stage of the cascade establishes a bias towards visual information that is relevant to the task (i.e., the ink colour) and involves the posterior part of the dorsolateral prefrontal cortex and the inferior frontal junction. The second step consists of maintaining the selected, task-relevant information in working memory; it involves the middle part of the dorsolateral prefrontal cortex. The third stage, response selection, resolves competition between potential responses; it involves the caudal part of the middle portion of the cingulate cortex. The fourth stage consists of response evaluation and feedback to the dorsolateral prefrontal cortex; this process involves the rostral part of the dorsal anterior cingulate cortex. The cascade-of-control model argues “that the degree of control that is exerted at earlier stages influence the degree of control that needs to be exerted at later stages” [29].

A network with a cascade-of-control architecture may prove resilient, i.e., adaptable, so that its function is preserved despite partial damage or dysfunction. As postulated in the model, adaptation and/or compensatory mechanisms may take place at different levels of control [29], involving processes such as higher levels of neural activity or recruitment of larger neuronal populations [32]. Based on the above findings, we formulated the following hypotheses:

- In healthy subjects, the neural activity elicited by Incongruent, Congruent or Neutral conditions is likely to differ between the first and the second parts of a prolonged Stroop task, particularly in the key regions of the cascade-of-control network.
- In post-COVID-19 patients, the neural activity elicited in the key regions of the cascade-of-control network by the Incongruent, Congruent and Neutral conditions is likely to differ from that in healthy subjects. Furthermore, the pattern of activity elicited by the Stroop effect may differ between individual patients.

We tested these hypotheses in a cross-sectional study by comparing activation patterns yielded by the Incongruent, Congruent and Neutral conditions of a colour–word Stroop task during the first and second parts of a prolonged test, both in healthy subjects and in patients in the aftermath of severe COVID-19. The present pilot study revealed largely preserved performance in all patients despite their history of severe COVID-19 infection. The maintenance of normal function was associated with two different patterns of activation, which are indicative of two distinct resilience profiles.

## 2. Materials and Methods

### 2.1. Participants

Six consecutive patients (four male, two female; age range 42–67 years) participated in this pilot study 9 months after having severe COVID-19 (Table 1). Inclusion criteria were COVID-19 diagnosed by PCR necessitating Intensive Care Unit stay and mechanical ventilation. Exclusion criteria were pre-existing neurocognitive impairment; history of traumatic brain injury, psychiatric, oncological and/or neurological disease; COVID-19-related stroke or cardiac arrest; colour-blindness; reading difficulties; medication affecting cognition; and conditions contraindicating MRI. All patients held full-time gainful employment before suffering from COVID-19. The control population consisted of 24 age-matched healthy subjects, 12 younger (mean age: 49 years; SD 5.2 years) and 12 older (mean age: 61.7 years; SD 3.8 years). Exclusion criteria for control subjects were the same as for patients. All patients and control subjects were right-handed, had normal vision and reported sufficient sleep preceding testing (patients: ≥7 h; control subjects: mean 7.2 h; SD 0.8 h). Patients and control subjects were examined during the same time period so that they did not differ in terms of psychological impact of the epidemic.

All patients and control subjects gave informed written consent according to procedures approved by the Ethics Committee of the Canton de Vaud.

All participants completed standardised scales and questionnaires prior to fMRI scanning, and Stroop task-associated mental fatigue was evaluated with an ad hoc visual analogue scale before and after fMRI (Table 1).

### 2.2. Stroop Task

The modified colour–word Stroop task [41], performed during fMRI scanning, comprised two parts (8 min each) programmed with E Prime 2.0 (Psychology Software Tools https://pstnet.com/products/e-prime/ accessed on 1 February 2018). Participants indicated the colour of test items (red, blue or yellow with a choice, per button press, between two response options shown at the top and bottom of the screen, respectively) in each of 3 conditions—Incongruent (ink colour different from word meaning), Congruent (ink colour the same as word meaning) and Neutral (ink colour of a rectangle). For each condition and part (40 presentations/part), accuracy and mean response times were calculated. The screen background was black, correct responses were equally attributed to the top and bottom buttons and the order of conditions was the same across all participants. Performance was assessed for accuracy, response times and the Stroop effect (i.e., the difference in response times in the Incongruent minus the Congruent condition, normalised to the mean of Incongruent, Congruent and Neutral conditions, in %). In the control population, performance was analysed with a mixed-design ANOVA, with group (Young, Old) as the between-subject factor and condition (Incongruent, Congruent, Neutral) and part (first, second) as the within-subject factors. The performance of individual patients was compared to the healthy range, defined as mean ± 2 standard deviations of the control population.

### 2.3. fMRI Data Acquisition and AnalysisStroop Task

MRI data sets were collected with a 3T Siemens Magnetom Prisma scanner with a 64-channel headcoil at the Lemanic Biomedical Imaging Center, Centre Hospitalier Universitaire Vaudois, Lausanne. Functional MR images were acquired with a multiband-2 echo planar imaging gradient echo sequence, and structural MR images included a high-resolution T1-weighted 3D gradient echo sequence. Both types of images were analysed using Statistical Parametric Mapping software (SPM12, Wellcome Department of Cognitive Neurology, London, UK) and standard preprocessing as described previously [42].

For each participant, first-level statistics used a GLM, including the realignment parameters as regressors and contrasts of interest for each condition (Incongruent, Congruent and Neutral conditions for Part 1 and Part 2). All fMRI data were restricted to voxels with a probability of >50% to belong to grey matter, as defined in the a priori template in SPM12. For the control group, second-level (group-level) statistical analyses were based on the random field theory. First, for the control subjects, the impact of age on activation patterns was analysed with a general mixed-design ANOVA with group (Young, Old) as the between-subject factor and condition (Incongruent, Congruent, Neutral) and part (Part 1, Part 2) as the within-subject factors. The second analysis, in which all control subjects were considered together (i.e., without separating Young and Old subjects), evaluated the activation patterns elicited by the Stroop effect across task exposure with the interaction of condition (Incongruent, Congruent, Neutral) × part (Part 1, Part 2). The third analysis on neuroimaging data evaluated the Stroop effect by means of a post hoc comparison of Incongruent and Congruent conditions for each part separately in the group of control subjects as well as in individual control subjects and patients.

## 3. Results

### 3.1. Control Population

All control subjects performed the Stroop task without difficulties. Accuracy and response times were analysed with a mixed-design ANOVA with group (Young, Old) as the between-subject factor and condition (Incongruent, Congruent, Neutral) and part (Part 1, Part 2) as the within-subject factors.

Accuracy was at or near ceiling level except for the incongruent condition during Part 1 (Table 2). It yielded a significant condition × part interaction (F (1,22) = 14.487; *p* = 0.001) and significant main effects for the factors condition (F (1,22) = 51.908; *p* < 0.001) and part (F (1,22) = 24.548; *p* < 0.001). All these effects were driven by an improvement in the Incongruent condition in Part 2. The interactions of condition × part × group (F (1,22) = 0.004; *p* = 0.947), condition × group (F (1,22) = 0.206; *p* = 0.654) and part × group (F (1,22) = 0.085; *p* = 0.773) as well as the main effect of group (F (1,22) = 0.038; *p* = 0.471) were not significant.

Response times (Table 2) yielded a significant condition × part interaction (F (1,22) = 10.689; *p* = 0.004, driven by a faster Incongruent condition during Part 2) and significant main effects of condition (F (1,22) = 231.433; *p* < 0.001, slower performance during Incongruent condition), part (F (1,22) = 69.514; *p* < 0.001, faster performance during Part 2) and group (F (1,22) = 4.627; *p* = 0.043, slower performance in Old group). The interactions of condition × part × group (F (1,22) = 0.386; *p* = 0.541), condition × group (F (1,22) = 1.653; *p* = 0.212) and part × group (F (1,22) = 2.719; *p* = 0.113) were not significant.

The Stroop effect was assessed by the difference in response times of the Incongruent vs. Congruent condition, normalised to the mean of response times of the Incongruent, Congruent and Neutral conditions (in %). It was analysed with a general mixed-design ANOVA with group (Young, Old) as the between-subject factor and part (Part 1, Part 2) as the within-subject factor. It yielded a significant main effect of part (F (1,22) = 8.664; *p* = 0.008; this was due to a decrease in Stroop effect-associated activity in Part 2). The interaction of part × group (F (1,22) = 0.002; *p* = 0.964) as well as the main effect of group (F (1,22) = 1.067; *p* = 0.313) were not significant.

Activation patterns were analysed with a general mixed-design ANOVA, with group (Young, Old) as the between-subject factor and condition (Incongruent, Congruent, Neutral) and part (Part 1, Part 2) as the within-subject factors. The three-way interaction of group × condition × part yielded small clusters on the right side in the lingual, superior temporal and middle temporal gyri, putamen and cerebellum (Table 3). The interaction of group × condition yielded significant clusters in the left and right cerebellum. It is to be noted that these small, isolated clusters were outside the Stroop network. The interaction of group × part did not yield any significant clusters, nor did the main effect of group.

**Figure 2:**
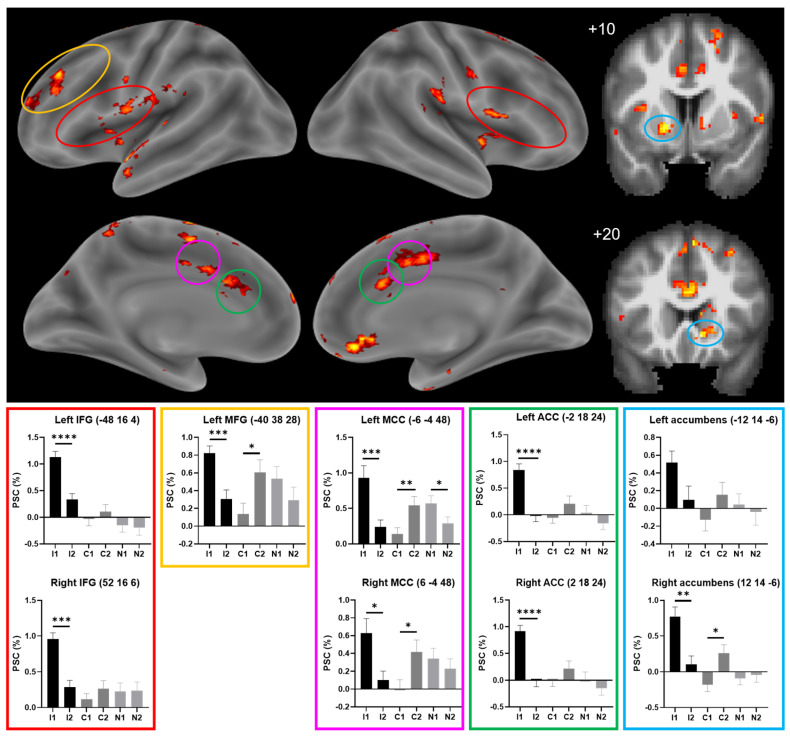
**Top**: Surface renderings (lateral and medial views of left and right hemispheres) and coronal sections (+10, +20; left on the left) of activation showing in healthy subjects a significant interaction in the mixed-design ANOVA with condition and part as the within-subject factors. All maps thresholded at *p* < 0.01 and with a cluster extent of k > 17. Key regions of the cascade-of-control model are circled in colour (same code as in Figure 1, with MNI coordinates): inferior frontal gyrus in red; middle frontal gyrus in yellow; middle cingulate cortex in purple; and anterior cingulate cortex in green. In addition, nucleus accumbens is highlighted, circled in blue. **Bottom**: Graphs (mean and SEM) of the percentage of BOLD signal changes for Incongruent (I), Congruent (C) and Neutral (N) conditions during Part 1 and Part 2 in the key regions of the cascade-of-control model (outlined in colour; [29]) and in the nucleus accumbens. One (*) to four (****) asterisks mark a significant difference in activation during the Incongruent condition and during the Congruent condition in Part 1 vs. Part 2 (*t* test, *p* < 0.05/0.01/0.001, respectively; Bonferroni corrected by ROI). ACC: anterior cingulate cortex; IFG: inferior frontal gyrus; MCC: middle cingulate cortex; MFG: middle frontal gyrus.

The interaction of condition × part yielded significant clusters bilaterally in the inferior frontal gyrus extending to the inferior frontal junction, the anterior part of the insula, the parietal operculum, the middle part of the cingulate cortex, the dorsal part of the anterior cingulate cortex and the nucleus accumbens as well as within the left hemisphere in the medial frontal gyrus and within the right hemisphere in the ventro-medial prefrontal cortex (Figure 2 top).

Within the key regions of the cascade-of-control model, the effect was driven by two effects (Figure 2 bottom). First, within the inferior frontal gyrus bilaterally, the left middle frontal gyrus, the middle cingulate cortex bilaterally, the anterior cingulate cortex bilaterally and the right nucleus accumbens, the effect was driven by (i) greater activity elicited by the Incongruent than the Congruent or Neutral conditions during Part 1; and (ii) greater activity elicited by the Incongruent condition in Part 1 than in Part 2. A post hoc comparison (Bonferroni corrected by ROI) of activation during the Incongruent, the Congruent and the Neutral conditions in Part 1 vs. Part 2 revealed a significant decrease in the Incongruent condition in these eight regions (Figure 2 bottom). Second, in the left middle cingulate cortex and the right nucleus accumbens, the effect was driven by lesser activity elicited by the Congruent condition in Part 1 than in Part 2. A post hoc comparison (Bonferroni corrected by ROI) of activation during the Incongruent, the Congruent and the Neutral conditions in Part 1 vs. Part 2 revealed a significant increase in the Congruent condition in these two regions (Figure 2 bottom).

**Figure 3:**
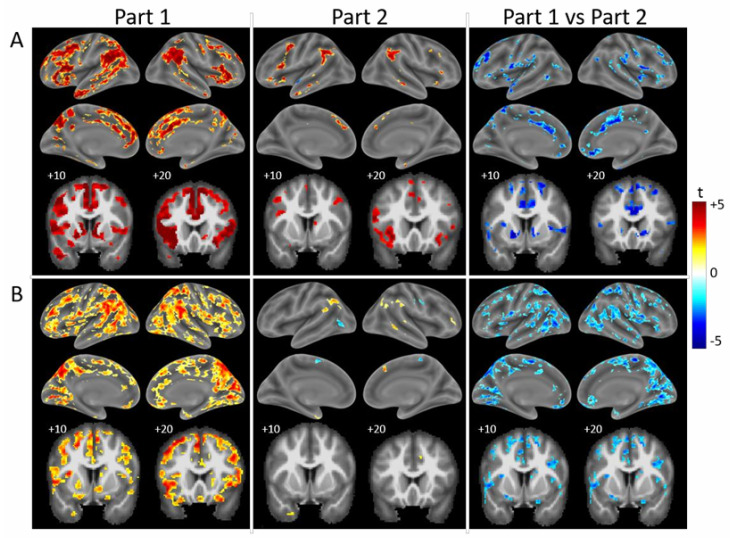
Surface renderings (lateral and medial views of left and right hemispheres) and coronal sections (+10, +20; left on the left) of activation patterns elicited by the comparison of Incongruent vs. Congruent during Part 1 (left column), Part 2 (middle column) and the change between Part 1 and Part 2 (right column; clusters with Part 1 > Part 2 are in cold colours; clusters with Part 1 < Part 2 are in warm colours) in healthy subjects. (**A**) Group analysis of 24 control subjects. Maps thresholded at *p* < 0.01 and cluster extent of k > 27. (**B**) Typical control subject (C19). Maps thresholded at *p* < 0.05 and cluster extent of k > 62.

A post hoc analysis of Part 1 revealed greater activity elicited by the Incongruent than the Congruent condition bilaterally within large parts of the fronto-parieto-temporal cortex, including in the key regions of the cascade-of-control model, i.e., the inferior frontal gyrus, the middle frontal gyrus and the middle and the anterior cingulate cortex, as well as in the nucleus accumbens (Figure 3A).

During Part 2, greater activity was elicited by the Incongruent than the Congruent condition in small clusters in the inferior frontal gyrus, the middle frontal gyrus and the anterior cingulate cortex and/or the adjacent medial frontal cortex, as well as bilaterally in the inferior parietal lobule and in small clusters on the temporal convexity. The comparison of the contrast Incongruent > Congruent during Part 1 vs. Part 2 yielded clusters of a significant decrease in the inferior frontal gyrus (extending to the inferior frontal junction), the middle frontal gyrus, the middle and anterior cingulate cortex and the nucleus accumbens, as well as the temporo-parietal junction, the praecuneus and parts of the temporal convexity. These clusters tended to include regions with a significant interaction of condition × part (shown in Figure 2). Very similar patterns were observed in individual control subjects, of whom a typical example is shown in Figure 3B.

### 3.2. Patient Population

All patients reported fatigue in both the motor/physical and cognitive/mental domains (Table 1). Compared to our control population, fatigue was rated predominantly as severe or moderate in four patients (P1, P2, P3, P4) and as moderate or mild in two (P5, P6). The Hospital Anxiety and Depression Scale highlighted symptoms of depression in two patients (P1, P2) and anxiety in two (P2, P6). The French Dimensional Apathy Scale showed symptoms in the executive range in five patients (P1, P2, P4, P5, P6) but none in the emotion or initiative ranges. The Perceived Stress Scale indicated a high level of stress in one patient (P1). The Epworth Sleepiness Score yielded abnormal scores in none of the patients and the Insomnia Severity Score in one (P6). The Quality of Life after Brain Injury Questionnaire yielded abnormally low scores in two patients (P1, P2). The Big Five Personality Test highlighted that the two factors with the highest scores were Conscientiousness and Agreeableness in five patients (P1, P3, P4, P5, P6, a similar profile to the control population) and Conscientiousness and Neuroticism in one (P2).

**Figure 4:**
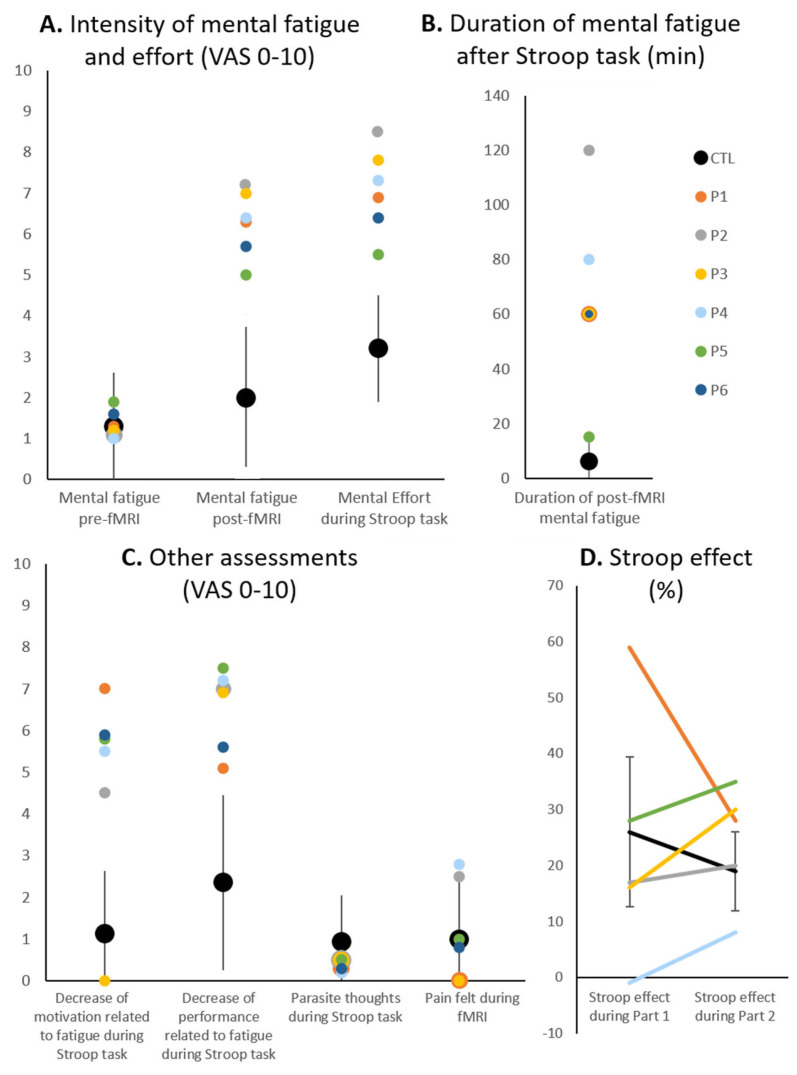
(**A**) Perceived mental fatigue before and after the fMRI paradigm; perceived mental effort during the Stroop task. (**B**) Duration of perceived mental fatigue after the experimental paradigm. (**C**) Perceived decrease in motivation and performance related to fatigue during the Stroop task, parasite thoughts occurring during the Stroop task and pain felt during the fMRI paradigm. (**D**) Stroop effect as assessed by the difference in response times between the Incongruent and Congruent condition, normalised to the mean of response times of the Incongruent, Congruent and Neutral conditions (in %). Black dots (**A**–**C**) and black line (**D**) indicate mean and vertical grey lines standard deviation of scores of the control population (CTL), colour dots and lines those of individual patients.

Mental fatigue as assessed before the fMRI paradigm was comparable in control subjects and in patients (Figure 4A). After the fMRI paradigm, all patients reported high levels of mental fatigue, which were outside the range of control subjects (outside two standard deviations of the mean of the control population) for four patients (P1–P4). Their perception of mental effort during the Stroop task was also higher than that of controls except for P5. The duration of mental fatigue induced by the Stroop task was higher in patients than in controls except for P5 (Figure 4B). The perception of a decrease in motivation related to fatigue during the Stroop task was outside the range of control subjects for five patients (P1, P2, P4–P6) and the perception of a decrease in performance related to fatigue during the Stroop task was outside the range of control subjects for four patients (P2–P5), whereas the presence of parasite thoughts during the Stroop task and the pain felt during the fMRI paradigm were comparable to those of the controls (Figure 4C).

All patients performed the Stroop task without major difficulties (Table 2). Accuracy was at or near ceiling level with the exception of the Incongruent condition during Part 1. Response times tended to be within the range of two standard deviations to the mean of the control population for all conditions with the exception of the Incongruent condition in P1. The Stroop effect was assessed by the difference in response times between the Incongruent and Congruent conditions, normalised to the mean of response times of the Incongruent, Congruent and Neutral conditions. Unlike in healthy subjects, it tended to increase between Part 1 and Part 2 in most patients with the exception of P1, who had an exceptionally high Stroop effect during Part 1 (Figure 4D).

**Figure 5:**
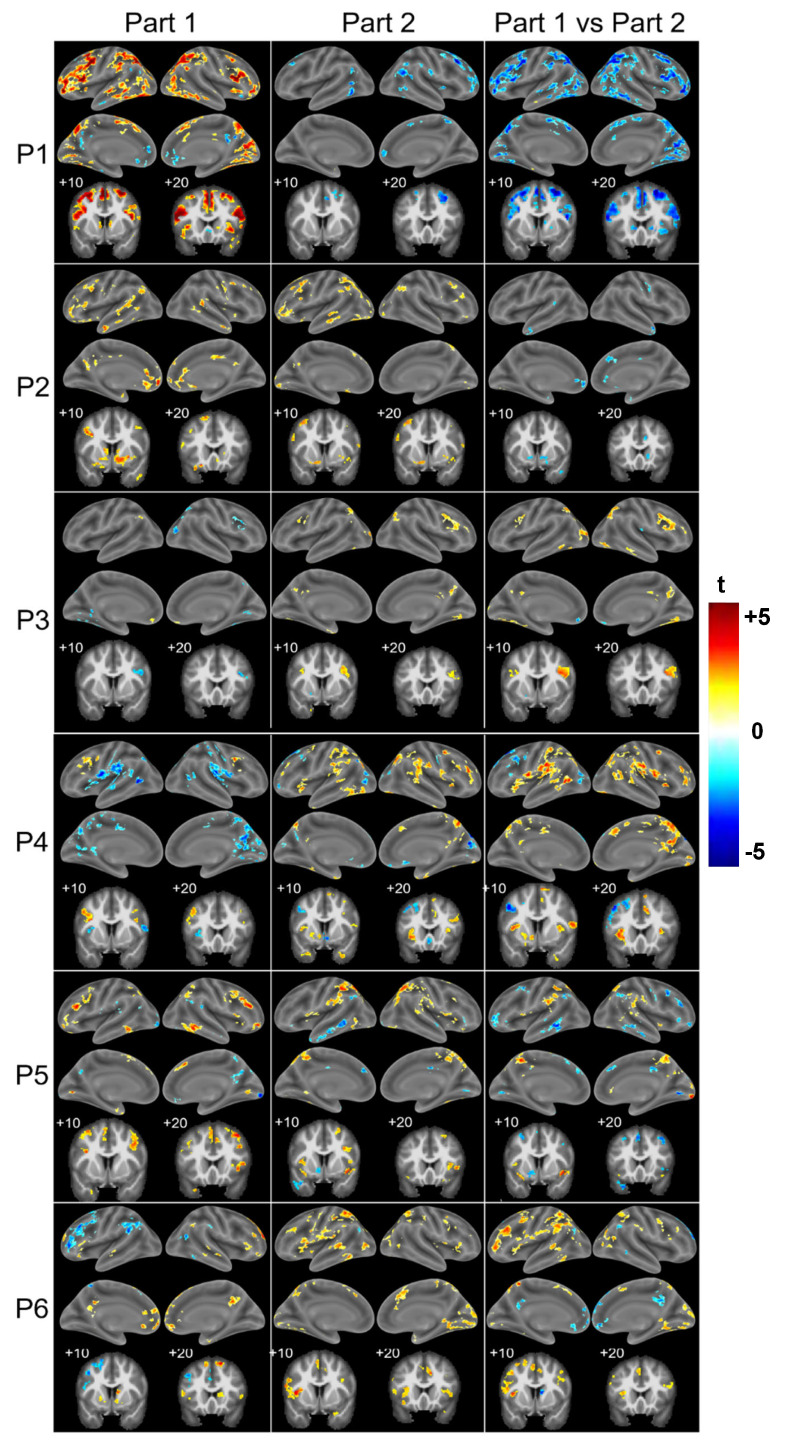
Surface renderings (lateral and medial views of left and right hemispheres) and coronal sections (+10, +20; left on the left) of activation patterns elicited by the comparison of Incongruent vs. Congruent during Part 1 (**left** column) and Part 2 (**middle** column), and the change between Part 1 and Part 2 (Part 2 minus Part 1, **right** column) in individual patients. Maps thresholded at *p* < 0.05 and cluster extent of k > 62.

The Stroop effect, assessed as Incongruent > Congruent, elicited activation that differed between patients and highlighted two global patterns (Figure 5). One subgroup of patients (P1, P2, P5) tended to present greater activation by the Incongruent than Congruent condition during Part 1. This was the case in the bilateral inferior frontal gyrus (P1, P2, P5), in the left middle frontal gyrus (P1, P2 et P5), in the bilateral middle cingulate and/or the adjacent medial frontal cortex (P1, P2), in the right anterior cingulate and/or the adjacent medial frontal cortex (P1, P2 et P5), in the left anterior cingulate cortex (P2) and in the bilateral nucleus accumbens (P1, P2). During Part 2, the activity yielded by the contrast Incongruent > Congruent decreased significantly as compared to Part 1 in the bilateral inferior frontal gyrus (P1), in the left middle frontal gyrus (P1, P5), in the bilateral or the right middle cingulate and the adjacent medial frontal cortex respectively (P1, P2), in the right anterior cingulate and/or the adjacent medial frontal cortex (P1, P2, P5) and in the bilateral nucleus accumbens (P1, P2).

In the other subgroup (P3, P4, P6), the contrast Incongruent > Congruent yielded only a few activation clusters during Part 1 (Figure 5): P3 had none in the target regions, P4 in one region (left middle frontal gyrus) and P6 in three of the nine regions (bilateral inferior frontal gyrus and left anterior cingulate cortex). During Part 2, the activity elicited by the contrast Incongruent > Congruent tended to increase, as seen in all three patients in the bilateral inferior frontal gyrus and in individual patients in the left nucleus accumbens and in the right anterior cingulate and/or the adjacent medial frontal cortex (P4) and in the left middle frontal gyrus (P6). The only decrease in the contrast of Incongruent > Congruent occurred in the left middle frontal gyrus in one patient (P4).

An analysis of the patient group as a whole did not reveal any significant changes between Part 1 and Part 2 for activation patterns elicited by the contrast of Incongruent minus Congruent. The same criteria were used as in healthy subjects (maps thresholded at *p* < 0.01 and cluster extent of k > 27), where this comparison yielded clusters of a significant decrease within the key regions of the cascade-of-control model (Figure 3A).

**Figure 6:**
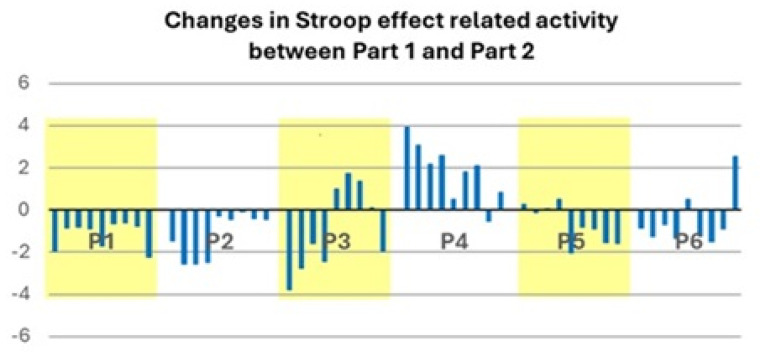
Graph showing the percentage of BOLD signal changes in the contrast of Incongruent minus Congruent condition between Part 1 and Part 2 in the key regions of the cascade-of-control model. For each individual patient (P1–P6) the 9 ROIs are shown from left to right: left IFG, right IFG, left MFG, left MCC, right MCC, left ACC, right ACC, left nucleus accumbens, right nucleus accumbens. Same abbreviations and MNI coordinates as in Figure 2. ROIs of P1, P3 and P5 are on yellow and those of P2, P4 and P6 on white background.

Changes in activation profiles within the seven key regions of the cascade-of-control model (bilateral inferior frontal gyrus, middle and anterior cingulate cortex, left middle frontal gyrus) and in bilateral nucleus accumbens, which occurred between Part 1 and Part 2 (Figure 6), differed between patients and separated the patient population into two subgroups. One subgroup (P1, P2, P5) presented between Part 1 and Part 2 a decrease in activation by the Incongruent vs. Congruent condition in the key regions (P1 and P2 in all nine, P5 in seven, with minor increases in two regions). The other subgroup (P3, P4, P6) presented a marked increase in activation by the Incongruent vs. Congruent condition in eight (P4), four (P3) and two regions (P6).

## 4. Discussion

### 4.1. Adaptation During a Prolonged Task in Healthy Subjects

In healthy subjects, the key regions of the cascade-of-control model presented across the two parts of the Stroop task two distinct sequences of activation. These differences may reflect plasticity within two distinct neural networks.

#### 4.1.1. Salience Network

The first sequence was present bilaterally in the opercular part of the inferior frontal gyrus, in the anterior cingulate cortex and in the right nucleus accumbens, where the Incongruent condition elicited greater activity than the Congruent and Neutral conditions during Part 1 or than any condition during Part 2. In other words, the greater demand on neural activity during the Incongruent condition, as compared to the Congruent or Neutral conditions, subsided during the second part of the 16 min Stroop task. We interpret this as a sharpening of the neural population involved in the Stroop effect through training, similar to what has been reported for learning-induced plasticity in spatial representations (e.g., [43]).

The opercular part of the inferior frontal gyrus and the anterior cingulate cortex are part of the salience network [44], the role of which was specifically pointed out in respect to the Stroop task [45]. Defined on the basis of resting-state connectivity, the salience network comprises, along with the dorsal anterior cingulate cortex and the opercular part of the prefrontal cortex, the anterior insula and the inferior parietal lobule [46,47,48,49]. In our pilot study, all four regions yielded a significant interaction of condition × part (Figure 2). The third region which shares the same response pattern, the nucleus accumbens, is not named in the cascade-of-control model [29]; it is known, however, to be involved in salience and reward/motivational processing [50].

#### 4.1.2. Top-Down Control Network

The second sequence was present in the left middle frontal gyrus and bilaterally in the middle cingulate cortex. The cascade-of-control model [29] posits that during the Stroop paradigm, the left middle frontal gyrus maintains the relevant information in short-term memory [51,52], in agreement with other findings highlighting the role of the middle frontal gyrus in working memory and in training-related plasticity [29]. According to the model, the middle cingulate cortex plays a role in response selection [51,52,53,54]. Interestingly, both regions, the middle frontal gyrus and the middle cingulate cortex, participate in a large network, which is defined on the basis of resting-state connectivity and which is believed to be involved in top-down control [46,55]. In these two regions, there was a decrease in neural activity between Part 1 and Part 2 for the Incongruent condition. This decrease in neural activity elicited by the Incongruent condition may reflect a training-induced sharpening of the neural population involved in the Stroop effect, similar to what we observed in the inferior frontal gyrus and the anterior cingulate cortex.

The increase in neural activity elicited by the Congruent condition between Part 1 and Part 2 in the left middle cingulate cortex and the right nucleus accumbens may be representative of costs related to a prolonged, albeit easy, task. The Congruent condition requires less mental effort than the Incongruent. Over a long period it may, however, require greater top-down attentional and motivational control to maintain a satisfactory level of performance [56]. This may lead to greater activity in the left middle cingulate cortex, which is involved in top-down control and which plays a role in response selection [29], and in the right nucleus accumbens, which is involved in motivational and salience-related behaviour [57].

#### 4.1.3. Effect of a Prolonged Stroop Task

A post hoc comparison of the Incongruent vs. Congruent conditions yielded different results in Part 1 and Part 2 (Figure 2). However, there was a great similarity between the four regions named in the cascade-of-control model (bilateral inferior frontal gyrus, left middle frontal gyrus, middle and anterior cingulate cortex) and the nucleus accumbens, both at the level of the control population and in individual control subjects (Figure 3). In the four regions of the cascade-of-control model plus in the nucleus accumbens, the Incongruent condition elicited greater activity than the Congruent condition during Part 1. The contrast of Incongruent > Congruent decreased significantly during Part 2 in all five regions.

### 4.2. Two Profiles of Network Resilience After Severe COVID-19

The patients who participated in this pilot study had suffered from severe COVID-19 which required Intensive Care Unit hospitalisation, but they sustained no detectable structural brain damage. They were thus different from previously investigated patients with flu-like symptoms, which did not require hospitalisation [58], patients with a mild form of COVID-19 [59] or patients suffering from long COVID [22] and post-COVID-19 condition [23]. Patients in our pilot study performed the Stroop task within limits of normal performance with the exception of one response time in one patient and one accuracy measure in another patient (Table 2). All patients reported similar complaints of fatigue with the exception of P5 (Figure 4). However, they differed with respect to activation patterns elicited during the Stroop task. The responsiveness of the key regions of the cascade-of-control model (left and right inferior frontal gyrus, left middle frontal gyrus, left and right anterior cingulate and/or adjacent medial prefrontal cortex, left and right middle cingulate and/or adjacent medial prefrontal cortex) and of the left and right nucleus accumbens followed two distinct profiles (a schematic representation of these nine locations is shown in Figure 6).

Three patients (P1, P2, P5) had similar activation profiles to those found in healthy subjects. During Part 1, the contrast of Incongruent > Congruent was positive (between four and nine locations of the nine involved: eight in P1, nine in P2, four in P5). Between Part 1 and Part 2, there tended to be a decrease in several of these locations (eight in P1, four in P2, two in P5) and never an increase in this contrast.

The other three patients (P3, P4, P6) differed substantially from healthy subjects. Their profile was characterised by the initial weakness of the contrast Incongruent > Congruent, with a subsequent tendency to increase. More specifically, during Part 1, the contrast of Incongruent > Congruent was rarely positive (one location in P4, three in P6) and was occasionally negative (one location each in P3 and P6, three in P4). Between Part 1 and Part 2 there was only once a decrease (one location in P4), whereas an increase occurred more frequently (two locations in P3, four in P4, three in P6). It is noteworthy that the region with the activation sequence which was nearest to the healthy situation, i.e., the left and right inferior frontal gyrus, was part of the salience network. Furthermore, it is striking that the most abnormal locations, the anterior cingulate cortex, the middle cingulate cortex and nucleus accumbens on either side, are known to be interconnected [60].

Our results indicate that there are two distinct functional correlates of the severe form of COVID-19. At the individual patient level, the contrast of Incongruent > Congruent tended to be (i) similar to that of healthy subjects; or (ii) weaker than in healthy subjects with s subsequent tendency to increase. The two activation profiles which we identified in individual post-COVID-19 patients, similar-to-healthy and the weaker-than-healthy, were found in association with fatigue in other conditions (for review, see [61,62]).

### 4.3. Cognitive Performance and Functional Correlates in the Aftermath of COVID-19

Cognitive performance, including on the Stroop test, as well as neural correlates have been investigated in patients who suffered from COVID-19.

Specific deficits have been described in association with long COVID. A behavioural study using the colour–word Stroop task was used to explore cognitive conflict by comparing the impact across stimulus presentations [63]. Response times to the target stimulus—Incongruent, Congruent or Neutral—was shown to be impacted by the preceding condition. Patients with long COVID as compared to healthy controls had significantly longer response times. More generally, long COVID appears to be associated with executive dysfunction and disruptions in frontal and cerebellar regions [64].

An fMRI study investigated functional connectivity between brain regions belonging to salience or default mode network hubs while subjects performed the colour–word Stroop task [22]. Patients with long COVID had stronger connections between the rostral medulla and two other regions, the midbrain and a default mode hub, whereas other connections tended to be weaker.

Post-COVID-19 condition and fatigue are accompanied by changes in neural activity, as demonstrated with working memory tasks in two recent studies. Patients with post-COVID-19 condition presented greater activation than controls across the working memory network during a two-back (but not zero- or one-back) version of the task; the difference was due to a greater task-related increase in the superior frontal gyrus and a lesser decrease in the default mode network [23]. A second study, using a two-back task, reported that patients who suffered from post-COVID-19 fatigue, as compared to healthy controls, had a stronger, bilateral activation of the middle frontal gyrus [24].

Cognitive dysfunction also affects patients who have suffered from mild to moderate COVID-19 which did not require hospitalisation, as demonstrated in two recent cross-sectional studies. They were found to have longer response times on the Stroop task as well as in other cognitive tasks at 3–6 months after disease onset [65] and at 28 months [66].

Interestingly, not only patients who suffered from COVID-19 but also those who experienced bereavement during the COVID-19 epidemic due to the death of a friend or close associate were shown to suffer from cognitive dysregulation, in particular of affective attentional processes [67].

### 4.4. Variety of Resting-State Functional Connectivity Profiles in COVID-19

Individual studies depicted differences which were partially related to the severity of the SARS-CoV-2 infection and the time since onset. The mild form of COVID-19 was found to be associated with alterations in resting-state functional connectivity in a longitudinal study that compared the same participants before and after SARS-CoV-2 infection [59] but not in a cross-sectional study 3 months after the onset of the disease [68]. Mild to moderate COVID-19 infection was shown to be associated in the chronic stage with lower functional connectivity in multiple brain regions [69]. Flu-like symptoms which did not require hospitalisation yielded decreased functional connectivity 3 days [58] as well as 4–5 months after a positive SARS-CoV-2 test [70]. COVID-19 which required hospitalisation was associated with enhanced functional connectivity in the acute stage [71] as well as at 6 months [72,73]. Severe COVID-19 which required Intensive Care Unit hospitalisation yielded at 1–5 months abnormal resting-state functional connectivity, which was correlated with the systemic immune–inflammation index [33]. Critically ill patients with disorders of consciousness presented a widespread decrease in structural and functional connectivity [74].

A comparison across degrees of severity during follow-up at 6–9 months highlighted disturbances of resting-state functional connectivity after moderate to severe, but not mild, COVID-19 [25] as well as frequent association with cognitive impairment [26,27] of resting-state functional connectivity; a decrease was reported 11 months after a mild, moderate or severe SARS-CoV-2 infection [75] and an increase 1.8 years after a mild or moderate infection [76].

### 4.5. Comparison with Activation Patterns in Chronic Fatigue Syndrome

fMRI studies in patients suffering from chronic fatigue syndrome have reported task-related increases in activations and recruitment of additional brain regions by cognitive tasks investigating working memory, attention, reward and motivation, sensory information processing or emotional conflict. As pointed out in systematic reviews, when tasks with increasing load or complexity were used, decreased activation in task-specific regions were reported (for review, see [61,62]). Individual studies show a complex picture. When the colour–word Stroop task was used, patients with chronic fatigue syndrome were as accurate as healthy controls in their performance but presented longer response times and more extended activations than controls [77]. The PASAT (paced auditory serial addition task) yielded in patients with chronic fatigue syndrome an increase in activation and/or recruitment of additional brain regions [78,79]. Other tasks yielded similar activation patterns in patients with chronic fatigue syndrome and in healthy controls. This was the case for motor imagery, which was associated with slower response times but overlapping activation patterns between patients with chronic fatigue syndrome and healthy controls [80].

Several studies reported lesser activation in patients with chronic fatigue syndrome than in healthy controls; this was the case for the activation of the auditory cortex during a fatigue-inducing auditory task [81]; of the basal ganglia, including the caudate nucleus, putamen and globus pallidus, during a monetary gambling task [82]; and in the left amygdala and midposterior insula during an emotional Stroop task [83].

### 4.6. Limitations

The relative paucity of COVID-19 cases included in this pilot study is partially due to the strict inclusion criteria we applied: ICU hospitalisation and no previous or COVID-19-related brain damage. The advantage of this approach is a homogeneous patient population. It is to be noted that the inclusion of patients was discontinued after the introduction of COVID-19 vaccination. As reported in a systematic review, pre-infection COVID-19 vaccination decreased the incidence of Intensive Care Unit admissions and the prevalence of post-COVID-19 symptoms [84]. This also changed the profile of patients who were hospitalised in Intensive Care Units; vaccinated patients who suffered from SARS-CoV2 infection and had severe disease tended to be older and to present more medical comorbidities than unvaccinated patients [85,86].

Our effect size calculations (based on Cohen’s method [87]), based on our patient and control populations, suggest that a minimum of 30 participants per group is required, and the ideal number is 300 to detect effects within each ROI. Thus, future studies need to include a very large number of participants. Specific approaches would be needed to analyse putative heterogeneity within the patient population, e.g., with principal component analysis.

The evidence from our homogeneous patient population highlights an important point, namely, that there are two distinct resilience profiles, which may be indicative of a propensity for further recovery and/or susceptibility to therapeutic interventions. It may also serve as basis for future study on neural mechanisms and lead to innovative therapeutic approaches [88].

Our approach is thus different from large-scale online studies of COVID-19 (e.g., [7,15,18]), which may eventually lead to patient health tracking and brain structure and function computation (e.g., [89,90,91]).

## 5. Conclusions

Considering neural networks, prior task-related activation studies in patients suffering from chronic fatigue syndrome were analysed at the group level and did not report on individual differences or different profiles of activation within their population [61,62,77,78,79,80,81,82,83]. To our knowledge, ours is the first pilot study reporting two types of activation patterns elicited by the Stroop task in a population which presents otherwise similar levels of fatigue. This raises two issues, which need to be addressed in further studies: the role of brain damage in post-COVID-19 fatigue and the recovery and response to therapeutic interventions.

Debilitating fatigue, which occurs in the aftermath of COVID-19, tends to be associated with difficulties in concentration and memory deficits; in many cases, however, in-depth neurological assessment failed to reveal brain lesions [2,21]. There are, however, indications of possible microscopic damage to brain tissue, which escapes standard clinical examinations [92]. Detailed structural analysis by means of diffusion-weighted MRI, including apparent fibre density, free water index and diffusion tensor imaging, carried out 3 months post-COVID-19 highlighted microstructural changes in the hemispheric grey and white matter. Positron emission tomography carried out at 12 months post-COVID-19 revealed different metabolic profiles in a group of seven patients with persistent neuropsychological deficits; glucose metabolism was normal in four patients but impaired in distinct brain regions in three others [93]. COVID-19-related fatigue and cognitive dysfunction may be linked to neuroinflammatory processes, which tend to persist over months after recovery from the acute disease [33,94,95]. COVID-19-induced microstructural changes, as described above, may interfere with the fine-tuning of neural networks, which underlie attention, motivation or executive control. High-demand cognitive work was shown to increase glutamate concentration the in prefrontal cortex (in healthy subjects [33]). The neuro-metabolic fine-tuning is likely to be affected by the decrease in glucose metabolism, which has been demonstrated in individual patients in the aftermath of COVID-19 [93].

The differences in task-related activation profiles which we observed in our post-COVID-19 patient population may offer insight into long-term outcomes and/or responsiveness to therapeutic interventions. Such a relationship has been demonstrated for several cognitive syndromes in the aftermath of stroke. In aphasia, activation patterns elicited by specific language tasks allow predictions as to subsequent recovery and, in some instances, to responsiveness to specific treatments (e.g., [96,97]). In neglect, the neural underpinning of a therapeutic intervention, adaptation to right-deviating prisms, has been elucidated by means of fMRI activation paradigms [98,99,100], and on the basis of these findings, responder profiles were defined [101,102,103,104].

Although the two types of activation profiles elicited by the Stroop task in our patient population may reflect different propensities for recovery, further investigations need to establish how patients with either activation profile respond to therapeutic interventions.

## Figures and Tables

**Table 1:** Patient (P1–P6) and control population characteristics; self-reported assessment by means of standardised scales, questionnaires and Stroop task-associated fatigue. For scales and questionnaires, bold denotes severe/abnormal, bold italics moderate and italics mild/near normal symptoms or scores.

	P1	P2	P3	P4	P5	P6	Controls Mean ± SD
Duration (days) of mechanical ventilation/ICU stay/acute hospitalisation/post-acute inpatient rehabilitation	22/25/36/17	18/21/40/53	17/21/29/14	11/12/24/0	50/67/82/37	17/21/29/31	-/-/-/-
**Standardised scales and questionnaires**							
Fatigue Scale for Motor and Cognitive Functions [33]: Motor 10–50/Cognitive 10–50	**41**/**40**	***30***/**35**	***28***/**34**	*26*/**36**	*23*/*27*	*26*/***28***	14.8 ± 3.6/16.0 ± 3.9
Brugmann Fatigue Scale [34]: Physical 0–12/Mental 0–12	**6** /**8**	**7** /**8**	***4***/**6**	***5***/**7**	***4/4***	***4***/***4***	1.0 ± 0.0/0.7 ± 0.5
Hospital Anxiety and Depression Scale [35]: Anxiety 0–21/Depression 0–21	6/**11**	**11**/*10*	5/4	3/1	0/2	*8* /0	5.1 ± 2.4/2.1 ± 1.8
French Dimensional Apathy Scale [36]: Executive 0–24/Emotion 0–24/Initiative 0–24	**14**/7/8	**15**/8/9	10/9/7	**15**/8/7	**14**/8/8	**13**/7/7	3.8 ± 1.6/3.3 ± 1.4/3.0 ± 1.4
Perceived Stress Scale [37]: 0–40	***20***	10	8	10	10	10	10.6 ± 5.7
Epworth Sleepiness Score [38]: 0–24	10	10	2	2	7	10	5.7 ± 4.1
Insomnia Severity Index [39]: 0–28	4	7	5	1	3	*8*	6.5 ± 4.6
Adapted Quality of Life after Brain Injury Questionnaire [40]: 0–100	*65*	*62*	95	84	77	78	83.6 ± 8.0
**Stroop task-associated fatigue (visual analogue scales: 1–10 or in minutes)**							
Mental fatigue pre-fMRI	1.3	1.1	1.2	1	1.9	1.6	1.3 ± 1.3
Mental fatigue post-fMRI	**6.3**	**7.2**	**7**	**6.4**	5	5.7	2 ± 2
Mental effort during Stroop task	**6.9**	**8.5**	**7.8**	**7.3**	5.5	**6.4**	3.2 ± 1.3
Motivation decrease related to Stroop-induced fatigue	**7**	**4.5**	0	**5.5**	**5.8**	**5.9**	1.1 ± 1.5
Performance decrease related to Stroop-induced fatigue	5.1	**7**	**6.9**	**7.2**	**7.5**	5.6	2.4 ± 2.1
Occurrence of parasite thoughts during Stroop task	0.3	0.5	0.5	0.2	0.5	0.3	0.9 ± 1.1
Pain felt during fMRI scanning	0	2.5	0	2.8	1	0.8	1 ± 1.4
Duration of Stroop-generated fatigue (minutes)	**60**	**120**	**60**	**80**	15	**60**	6 ± 7.9

**Table 2:** Performance of the control population (mean ± SD) and of patients (individual scores) in the colour–word Stroop task: accuracy (mean ± SD) and response times (mean ± SD) in Incongruent, Congruent and Neutral conditions during Part 1 and Part 2. Performance of control subjects for individual conditions were compared between Part 1 and Part 2 with a paired t-test. Patient performance outside the range of normal performance (defined as the mean ± 2 SD of the control population) is in bold.

	Accuracy (%)						Response Times (ms)					
	Incongruent		Congruent		Neutral		Incongruent		Congruent		Neutral	
	Part 1	Part 2	Part 1	Part 2	Part 1	Part 2	Part 1	Part 2	Part 1	Part 2	Part 1	Part 2
**Control population**												
	81 ± 14	94 ± 5	99 ± 2	100 ± 1	99 ± 3	100 ± 1	1636 ± 342	1466 ± 268	1282 ± 273	1232 ± 263	1184 ± 211	1107 ± 206
	*p* < 0.001		*p* = 0.185		*p* = 0.6162		*p* < 0.001		*p* = 0.010		*p* < 0.001	
**Patients**												
P1	75	95	100	100	100	100	**2471**	1918	1429	1466	1395	1406
P2	90	100	100	100	100	100	1327	1306	1136	1081	940	922
P3	90	100	100	100	100	**95**	1850	1844	1593	1387	1328	1356
P4	85	100	100	100	95	100	1606	1612	1621	1491	1355	1327
P5	90	100	100	100	100	100	1728	1599	1322	1145	1254	1168

**Table 3:** Brain regions showing significant effects in the three-way ANOVA (group × condition × part) and in the two-way ANOVA (group × condition).

Areas	Number of Voxels	Peak Intensity	Peak MNI Coordinates		
			x	y	z
**Three-way ANOVA (group × condition × part)**					
R lingual gyrus	33	9.20	12	−42	−2
R cerebellum (IV–V)	33	8.51	20	−50	−14
R superior temporal gyrus	31	8.44	50	−38	10
R putamen	51	7.80	28	6	−4
R middle temporal gyrus	19	7.76	46	−54	8
**Two-way ANOVA (group × condition)**					
L cerebellum (Crus 1)	30	9.74	−38	−82	−18
R cerebellum (VII)	26	7.14	40	−60	−42

## Data Availability

The raw data supporting the conclusions of this article is available upon request to the corresponding author.

## Supplementary Materials

The following supporting information can be downloaded at: https://www.mdpi.com/article/10.3390/brainsci15060635/s1, Table S1: Regions implicated in the Cascade-of-control model of the Stroop effect [29], as described in prior studies [41,51,52,53,54,105,106,107,108,109,110,111,112].
