# Supplementary material for: Resilience of Neural Networks Underlying the Stroop Effect in the Aftermath of Severe COVID-19: fMRI Pilot Study
Source: Brain Sci. 2025 Jun 12;15(6):635. doi: 10.3390/brainsci15060635 (PMC12191155; doi:10.3390/brainsci15060635)
Supplement: Supplementary file 1 [file brainsci-15-00635-s001.zip › brainsci-3612266-supplementary.pdf]

Table S1. Regions implicated in the Cascade-of-control model of the Stroop effect (Banich 2019), as described in prior studies. Coordinates reported in these studies and shown in Figure 1 are indicated here in MNI space (transformed if in Talairach in the original publication).

Publication	Paradigm	Contrast	Side	BA	x	y	z
**Establishing a bias towards task-relevant sensory or perceptual information: inferior frontal gyrus & junction**							
Banich et al. 2000, Cognitive Brain Research<br>Prefrontal regions play a predominant role in imposing an attentional set: evidence from fMRI [105]	Colour-word; Colour-object	Incongruent > Neutral	L	44	-50	-10	37
			R	44	55	18	37
			L	46	-44	31	22
			R	46	59	27	29
Banich et al. 2000, Journal of Cognitive Neuroscience<br>fMRI studies of stroop tasks reveal unique roles of anterior and posterior brain systems in attentional selection [41]	Colour-word > Spatial-word	Incongruent > Neutral	L	44	-48	7	29
			R	44	48	12	29
			L	46	-35	25	27
			R	46	44	25	27
Milham et al. 2001, Cognitive Brain Res<br>The relative involvement of anterior cingulate and prefrontal cortex in attentional control depends on nature of conflict [54]	Colour-word; Response-eligible and -ineligible incongruent conditions	Incongruent > Neutral	L	44/45	-35	22	27
Zysset et al. 2001, Neuroimage<br>Color-word matching Stroop task: separating interference and response conflict [106]	Colour-word	Incongruent > Neutral	L	44	-40	6	33
Milham et al. 2002, Brain and Cognition<br>Attentional control in the aging brain: insights from an fMRI study of the Stroop task [107]	Colour-word	Incongruent > Congruent & Neutral; Young	L	46	-50	48	10
			R	45/46	46	18	7
		Old	L	46	-46	44	10
			R	45/46	48	20	7
Mead et al. 2002, JINS<br>Neural basis of the Stroop interference task: response competition or selective attention? [108]	Colour-word	Incongruent > Neutral	L	44/6	-46	5	32
Norris et al. 2002, Neuroimage<br>An investigation of the value of spin-echo-based fMRI using a stroop color-word matching task and EPI at 3T [109]	Colour-word	Incongruent > Neutral	L	-	-40	5	36
			R	-	43	19	24
Potenza et al. 2003, American Journal of Psychiatry<br>An fMRI Stroop task study of ventromedial prefrontal cortical function in pathological gamblers [110]	Colour-word (Controls)	Incongruent > Congruent	R	-	47	9	38
Zysset et al. 2007, Neurobiology of Aging<br>Stroop interference, hemodynamic response and aging: an event-related fMRI study [111]	Colour-word	Incongruent > Neutral; Young & old	L	-	-46	19	31
			R	-	44	10	37

**Maintaining the relevant information in working memory: middle frontal gyrus**							
Milham et al. 2003, Cognitive Brain Res<br>Competition for priority in processing increases prefrontal cortex's involvement in top-down control: an event-related fMRI study of the Stroop task [51]	Colour-word;<br>Response-eligible and -ineligible incongruent conditions	Incongruent (eligible & ineligible) > neutral	L	46/9	-48	44	27
			R	46/9	52	42	27
Milham and Banich 2005, Human Brain Mapping<br>Anterior cingulate cortex: an fMRI analysis of conflict specificity and functional differentiation [52]	Colour-word	Congruent & incongruent > neutral	L	46	-44	46	22
			L	8	-30	26	54
			L	6	-46	7	57
			L	10	-29	46	3
			R	9	34	18	18
			R	10	35	31	22
			R	9	36	58	30
			R	8	28	46	44
			R	8	44	24	48
**Response selection: middle cingulate cortex**							
Milham et al. 2001, Cognitive Brain Res<br>The relative involvement of anterior cingulate and prefrontal cortex in attentional control depends on nature of conflict [54]	Colour-word;<br>Response-eligible and -ineligible incongruent conditions	Incongruent > neutral		32/6	1	12	48
		Incongruent-response eligible > incongruent-response ineligible	(R)	32/6	10	22	46
Milham et al. 2003, Cognitive Brain Res<br>Competition for priority in processing increases prefrontal cortex's involvement in top-down control: en event-related fMRI study of the Stroop task [51]	Colour-word;<br>Response-eligible and -ineligible incongruent conditions	Incongruent (eligible & ineligible) > neutral	R	32	10	33	31
Milham and Banich 2005, Human Brain Mapping<br>Anterior cingulate cortex: an fMRI analysis of conflict specificity and functional differentiation [52]	Colour-word	Incongruent > congruent	R	24	7	22	31
			L	32	-3	16	52
Liu et al. 2006, Cerebral Cortex<br>Functional dissociation of attentional selection within PFC: response and non-response related aspects of attentional selection as ascertained by fMRI [53]	Colour-word;<br>1 or 4 different words per condition	1-word > 4 word	R	32	5	25	44
			L	24	-7	5	40
**Evaluation of the response and feed-back: anterior cingulate cortex**							
Leung et al. 2000, Cerebral Cortex<br>An event-related functional MRI study of the Stroop color word interference task [112]	Colour-word	Incongruent > Congruent	L	32	-5	26	42
			R	32	7	26	43
Milham et al. 2002, Brain and Cognition<br>Attentional control in the aging brain: insights from an fMRI study of the Stroop task [107]	Colour-word	Incongruent > Congruent<br>Young	R	32	3	35	37
				32	5	20	44
